# Prevalence and diagnostic accuracy of different diagnostic tests for Chagas disease in an indigenous community of the Paraguayan Chaco

**DOI:** 10.1371/journal.pntd.0012861

**Published:** 2025-02-07

**Authors:** Sofia Ardiles-Ruesjas, Vidalia Lesmo, Valeria González-Romero, Zully Cubilla, Lilian Chena, Claudia Huber, María José Rivas, Patricia Saldaña, Adrián Carrascosa, Susana Méndez, Sergi Sanz, Sören L. Becker, Julio Alonso-Padilla, Irene Losada

**Affiliations:** 1 Barcelona Institute for Global Health (ISGlobal), Hospital Clínic, University of Barcelona, Barcelona, Spain; 2 Institute of Medical Microbiology and Hygiene, Saarland University, Homburg, Germany; 3 National Chagas Disease Control Program, Asuncion, Paraguay; 4 Teniente Irala Fernández Health Center, Teniente Primero Irala Fernández, Presidente Hayes, Paraguay; 5 Central Public Health Laboratory, Asunción, Paraguay; 6 Helmholtz Institute for Pharmaceutical Research Saarland (HIPS), Saarbrucken, Germany; 7 CIBER de Enfermedades Infecciosas, Instituto de Salud Carlos III (CIBERINFEC, ISCIII), Madrid, Spain; Fundacao Oswaldo Cruz, BRAZIL

## Abstract

**Introduction:**

Chagas disease (CD), caused by the protozoan *Trypanosoma cruzi (T. cruzi),* poses a major health challenge in Paraguay, especially in the resource-limited Chaco region. Rapid diagnostic tests (RDTs) are valuable tools to enhance diagnostic access. This study evaluates CD prevalence and risk factors in an indigenous community in the Paraguayan Chaco and validates the national RDT-based diagnostic algorithm for resource-limited settings against the recommended standard algorithm, which relies solely on conventional serological tests.

**Methodology:**

A descriptive cross-sectional study was conducted in Casanillo, Presidente Hayes, Paraguay. In July 2023, a two-week field campaign was executed using a non-probability convenience sampling method targeting individuals aged over 9 months. Screening involved a single RDT, with positives confirmed via enzyme-linked immunosorbent assay (ELISA). Algorithm accuracy was validated externally at the National Reference Laboratory of Paraguay against the standard algorithm, which, in this study, included an ELISA and Hemagglutination test. Discordant cases were resolved with a second ELISA or Immunofluorescence.

**Results:**

The study involved 999 participants, with a median age of 26 years (IQR 12-45), and 51.1% were female. The RDT-based diagnostic algorithm showed 97.1% agreement (κ = 0.94, 95%CI: 0.90–0.98) with the standard algorithm. The RDT alone had 96.0% agreement (κ = 0.91, 95%CI: 0.87–0.96), while the confirmatory ELISA had 94.3% agreement (κ = 0.88, 95%CI: 0.83–0.93). The algorithm’s sensitivity/specificity (95%CI) were 94.6% (89.2–97.8)/98.6% (96.1–99.7), with the RDT at 94.6% (89.2–97.8)/96.8% (93.6–98.7) and the ELISA at 96.9% (92.3–99.2)/92.7% (88.5–95.8). *T.cruzi* infection seroprevalence was 12.6% (95%CI: 9.56–16.52). Age, Sanapaná ethnicity, and awareness of CD vectors were significantly associated with infection odds. No significant associations were found with other typical CD risk factors, clinical history, or health habits.

**Conclusion:**

The study underscores the high burden of *T. cruzi* infection in indigenous communities in the Paraguayan Chaco, urging immediate interventions for improved diagnosis and treatment. The combination of RDTs with conventional serology for diagnostic screening in resource-constrained settings proved useful, and its further use is encouraged.

## Author summary

Chagas disease (CD) is a significant health issue in Paraguay, especially in the remote and resource-limited Chaco region. This disease, caused by the parasite *Trypanosoma cruzi (T. cruzi)*, is mainly spread by insects, and from mother to child, among others. Early and accurate diagnosis is crucial for effective treatment; however, conventional serological techniques require highly specific and extensive training as well as specialized equipment, which are often unavailable in resource-limited settings. Rapid diagnostic tests (RDTs) offer a practical solution as they are easy to use and provide quick results. In our study, we tested the diagnostic CD algorithm proposed by the Paraguayan National Clinical Guidelines for resource-limited areas, which combines the use of RDTs and traditional laboratory tests, in an indigenous community in the Chaco. We found that this approach was highly effective, with nearly perfect agreement with the reference algorithm. Our findings showed that 12.6% of the community members were infected with *T. cruzi.* We also identified infection-associated factors like age, ethnicity, and awareness of CD vectors. Our work confirms that the diagnostic algorithm recommended by Paraguayan health authorities is reliable and suitable for resource-constrained settings and further emphasizes the urgent need for targeted diagnosis and treatment programs in Chaco communities, which bear a heavy burden of CD.

## 1. Introduction

American trypanosomiasis or Chagas disease (CD), caused by the protozoan *Trypanosoma cruzi (T. cruzi)*, is considered to be responsible for the most significant morbidity and mortality among parasitic diseases in the world [1]. The disease is mainly transmitted by hemipteran insects of the subfamily *Triatominae* (bedbugs or *vinchucas*); but has alternative transmission pathways like orally through parasite-contaminated food or drink, and by vector-independent routes like blood transfusion and from mother-to-child [2].

According to the World Health Organization (WHO), between 6 and 7 million people are infected with *T. cruzi* worldwide and CD is considered a neglected tropical disease [3,4]. In the Americas, where CD is endemic in 21 countries, it is a serious public health problem and a constraining factor for the full economic and social development of the region [3,5]. It is estimated that around 70 million people are at risk of infection which leads to around 30,000 new cases and 12,000 deaths yearly [6]. The complex ecological interactions between parasites, vectors, and human populations, along with factors like inadequate surveillance, limited healthcare access, and lack of awareness, contribute to the ongoing challenge of controlling *T. cruzi* transmission [7,8].

In Paraguay, around 165,000 people are currently infected with *T. cruzi* and over 1.7 million are at risk of infection [9,10]. The establishment of comprehensive and inter-sectoral interventions for controlling CD have resulted in a substantial reduction in its incidence at national level [8,11]. A notable accomplishment includes the national-level certification of the interruption of *T. cruzi* intra-domiciliary transmission by *Triatoma infestans* vectors in 2018 [12]. Yet, recent epidemiological data suggest that the prevalence of *T. cruzi* infection in pregnant women ranges from 6% to 25% depending on the Department, and approximately 1.5% of blood donations nationally are infected with the protozoan [10].

The disease is distributed throughout Paraguay with different levels of prevalence, with the Chaco region (Departments of Presidente Hayes, Boquerón and Alto Paraguay) being the most endemic [13]. This geographical area represents 61% of the national territory and it is characterized by semi-arid to arid climate with limited access to water and extreme temperatures [14]. It is home to just over 2% of the country’s total population and has the highest concentration of ethnic groups and diversity of indigenous peoples (43% of the total, concentrating 13 of Paraguay’s 19 indigenous peoples [15].

Because of its social and ecological attributes, the Chaco is an area of significant concern for CD, and the most difficult to control effectively [13,16]. The results of serological and entomological surveys conducted across different communities in this area during the 1970’s to 1990’s revealed a high *T. cruzi* seropositivity, which ranged from 12 to 83% [13]. Although there is no recent information on seroprevalence in the same populations, these data suggest that challenges remain in access to treatment and diagnosis, and that the association between social determinants and disease incidence remains [16,17]

The natural progression of CD involves an initial acute phase marked by flu-like symptoms, leading to a chronic phase if untreated. Most individuals in this chronic phase remain asymptomatic throughout their lives. However, after 20–30 years around 30% will experience cardiac and/or digestive alterations [3]. Therefore, it is crucial that those infected have access to timely diagnosis and treatment to interrupt disease progression and prevent transmission [18]. However, access to diagnosis and medical care is far from universal. While there are no official global statistics available, it is estimated that less than 1% of those infected receive treatment, attributed to various factors, including the global under-diagnosis of the disease [7,19].

The diagnostic methodologies to detect the infection are stage dependent. In the acute stage direct detection of the parasite or its DNA content is sought due to the presence of detectable parasitemia. However, most detections are achieved in the chronic phase. Then, diagnosis relies on serological techniques for the identification of anti-*T. cruzi* circulating antibodies by means of enzyme-linked immunosorbent assays (ELISAs), indirect immunofluorescence (IIF) or hemagglutination inhibition (HAI) [20]. Due to the antigenic diversity of the parasite, the Pan American Health Organization (PAHO/WHO) recommends the agreement of two tests based on different antigens or principles to achieve a confirmed diagnosis, and the use of a third test in case of discordance [21].

Despite laboratory serological tests having good sensitivity and specificity[22], they require technically specialized personnel, equipment and infrastructure, which are scarce in most areas where the disease is endemic [7,20,23]. Even if available, residents of these areas may also face restricted access to health facilities and diagnostics due to economic barriers, such as associated costs and travel distances [24,25]*.* Moreover, recommended diagnostic and clinical management algorithms for CD often require multiple patient visits, which contributes to dropouts further hampering the access to treatment [26].

Responding to the need to establish more appropriate diagnostics for areas with limited resources, rapid diagnostic tests (RDTs) emerged as valuable alternatives [23,27]. They are easy to use, allow the processing at the point of contact independently from a cold chain, specific equipment and specialized technicians, and grant a quick turnaround of results (often in less than 30 minutes). Moreover, most RDTs do not require a large volume of sample and function with whole blood that can be obtained by finger prick, greatly simplifying logistics and increasing the willingness to undergo testing [23–27]. Many studies suggest that the accuracy of RDTs is good enough for screening individuals at risk from endemic areas [22–23]. In fact, this led to including the combination of an RDT plus a conventional assay for positive result confirmation as the diagnostic algorithm for detecting the chronic infection stage in rural and resource-constrained settings in the national guidelines of countries like Paraguay [10].

The purpose of this study was to evaluate the performance of the national diagnostic algorithm for *T. cruzi* chronic infections in adults in resource-constrained settings. This algorithm, which combines an RDT with one conventional serological test, was compared to the standard PAHO/WHO diagnostic algorithm, which relies on at least two conventional serological tests. Additionally, the study aimed to describe the seroprevalence and associated risk factors of CD in the indigenous community of Casanillo in the Paraguayan Chaco.

## 2. Methodology

### 2.1. Ethics statement

The study was performed in accordance with the principles of the Declaration of Helsinki. Its protocol was approved by the Ethics Committee of the Central Public Health Laboratory (CPHL) (ref.: CEI-LCSP No 262/301122; Asunción, Paraguay). Written informed consent was obtained from each participant or from the parent or legal guardian of children and adolescents aged below 18 years prior to participation.

All participants were immediately informed about their RDT results by a physician and were registered to enable the medical and nursery teams from the reference health care centers, to inform the result of the confirmation tests, guaranteeing all positively tested patients’ further medical evaluation and access to treatment. All personal information related to the sample was blinded until the result of the confirmatory serological test, and it was only revealed to the treating physician. In cases of discordant results, health personnel actively pursued patients to conduct additional testing to clarify their serological status. For minors with confirmed positive results and unknown maternal serological status, vertical transmission was investigated through active case search and mother screening to ensure comprehensive assessment and care. Likewise, if a mother tested positive, classic serological tests (e.g., ELISA) were conducted to confirm the diagnosis in her children as per national guidelines [10].

### 2.2. Study location and target population

**Fig 1:**
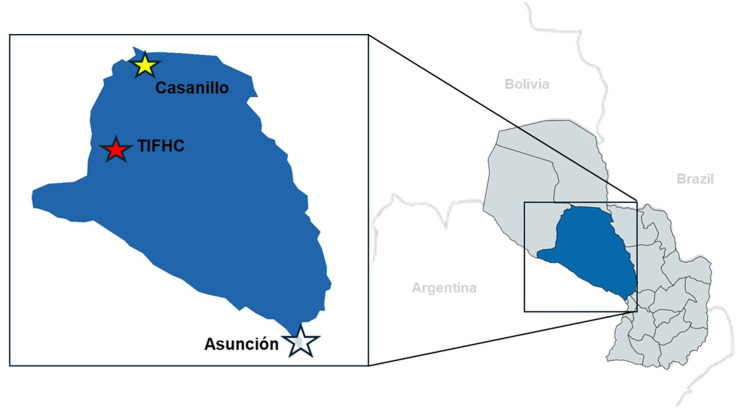
Geographic location of the President Hayes department in Paraguay and the study sites. The locations of the Casanillo community are represented by the yellow star and Teniente Irala Fernandez Health Center (TIFHC). Map adapted from Gabaldón-Figueira, JC et al. [28].

The geographic area defined for the interventions of this study is Casanillo, a rural indigenous community located in the north of the district of Teniente Primero Manuel Irala Fernández, in the northwest of the Department of Presidente Hayes, in the central area of the Chaco region. Casanillo is located 500 km away from Asunción (country capital), 300 km from Villa Hayes (departmental capital), and 66 km from Teniente Irala Fernandez Health Center (TIFHC), the closest reference advanced healthcare facility with a diagnostic laboratory (Fig 1). Due to challenging access and suboptimal road conditions, the journey from Casanillo to TIFHC takes up to two hours.

The community of Casanillo encompasses indigenous groups of the following ethnic families: Toba-Maskoy, Sanapaná, Guaná, Angaité, Ava, Guaraní, Enlhet Norte and Enxet Sur. The community has one primary health center, the Conamoctololac Primary Health Center (CPHC), which is staffed by a nurse and four health promoters.

According to the latest census dated June 2023 in Casanillo live 1,462 people divided in six villages: Casanillo (center) (n = 676), Campo Aroma (n = 248), Capiatá (n = 106), Linda Vista (n = 4), San Rafael (n = 280) and Tres Palmas (n = 148). For this study we also included the village of Campo Rayo (n = 47), which does not belong to Casanillo but is within the health service area of the CPHC. In total, 1,509 people were susceptible to being screened in the study.

### 2.3. Study design

In July 2023, a descriptive cross-sectional study was conducted over a two-week period through field screening campaigns. These were prearranged with community leaders and promoted by local health workers through information, education and communication (IEC) activities and community key actors. We employed a convenience non-probabilistic sampling method to all inhabitants aged >9 months, wherein a translator from the indigenous communities provided explanations and invitations to potential volunteers for participation in the study. Sampling was conducted for one to two days per village, varying the daily screening capacity between 60 and 100 individuals.

The field team, with local and National Chagas Control Program (NCCP) personnel, comprised eight people: four nurses, two medical doctors, and two nursing assistants. Among them, three people were assigned to enroll participants and obtaining informed consent, another three were responsible for performing and interpreting RDTs in situ, while two more were designated for collecting blood samples.

The following procedures were performed upon enrollment of each participant: 1) collection of data regarding personal, sociodemographic and clinical information, as well as epidemiological risk factors in a tailor-designed questionnaire upon enrollment; 2) RDT from finger-pricked whole blood was performed by trained personnel, and the results obtained were registered on site; 3) sampling of whole blood (~3–5 ml) by venous puncture to every individual with a positive RTD result and every one in three among the negatives; and 4) blood samples were transported daily in cooling boxes at 4ºC to the TIFHC laboratory for serum isolation, which was stored frozen until needed.

Due to cultural beliefs and local hesitancy toward blood extraction in the study population, the protocol was adapted to limit the number of venous blood samples taken, prioritizing those to confirm all RDT positive results and a representative subset (one-third) of all RDT-negative cases. Based on the formerly retrieved high sensitivity of the RDT used in the field [25,28], and thanks to a preparatory IEC community intervention, we managed to optimize community participation while balancing the need for diagnostic confirmation.

After initial diagnostic assessment at TIFHC, frozen samples were transported according to standard biosafety protocols to the CPHL, the nationwide reference laboratory in Asunción. The purpose was to validate the results of the diagnostic algorithm in resource-constrained settings (national algorithm) as recommended by the Paraguayan Clinical Guidelines by re-assessing the samples using two serological tests of different antigenic principles (standard algorithm) following the recommendation of the PAHO/WHO [10,21]. Moreover, an external control on TIFHC laboratory biochemist performance with CD diagnostics (ELISA tests) was made by repeating these tests at CPHL.

### 2.4. RDTs and serological methods

**Fig 2:**
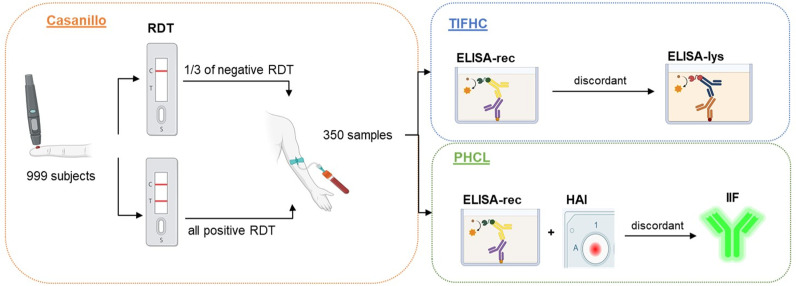
Schematic overview of the diagnostic workflow and sample validation process upon study participants enrolment.

Chagas Stat-Pak RDT (Chembio Inc., Medford, USA) was used for the field screening because of its availability, operational robustness, ease of use, and proved good performance in high to middle-high prevalence areas in the Southern Cone [24,26]. Moreover, it is in the portfolio of RDTs used by the NCCP. A parasite recombinant antigen ELISA (ELISA-rec; Wiener Chagatest v.3.0, Wiener Lab, Rosario, Argentina) was used as a confirmation test. When the results of these two techniques (RDT and ELISA) were positive, the sample was considered positive. In case of discordance between them, a second lysate ELISA test (ELISA-lys, IICS Chagas V2; Instituto de Investigación en Ciencias de las Salud, Asuncion, Paraguay) was used. At the CPHL in Asuncion, a HAI test (Wiener Lab) and the Wiener Chagatest v.3.0 ELISA were used as main serological tests. In cases of discordance, an in-house IIF test was used. These specific tests were chosen because they are the ones routinely employed at TIFHC and CLPH (Fig 2).

The RDT used finger-pricked whole blood as a sample. The other tests were conducted using serum samples. All assays were performed following the manufacturers’ instructions by trained personnel at TIFHC and CPHL laboratories. The RDT and IIF results were qualitative. The results from the ELISAs were quantitative; their output classified as negative or positive according to the optical density (OD) cutoff calculated following the manufacturers´ instructions. To reduce observer bias and ensure the validity of the study, the operators conducting the diagnostic tests were blinded to the results of previous assays. This blinding was achieved by assigning unique codes to sera samples and was consistently maintained across all assays.

### 2.5. Data collection

Study data were collected and managed using REDCap (Research Electronic Data Capture) hosted at the Institute of Global Health of Barcelona (ISGlobal), Spain [29,30]. REDCap is a secure, web-based software platform designed to support data capture for research studies, providing 1) an intuitive interface for validated data capture; 2) audit trails for tracking data manipulation and export procedures; 3) automated export procedures for seamless data downloads to common statistical packages; and 4) procedures for data integration and interoperability with external sources.

Data from the study participants, risk factors assessment, and the qualitative results of RDTs were collected on a paper questionnaire during the field intervention and later entered in REDCap. Similarly, laboratory results from the serological tests (ELISA, HAI, IIF) were also manually introduced in REDCap, or imported when available, for their result validation, safe storage and analysis.

### 2.6. Statistical analysis and study variables

The diagnostic accuracy assessment of the diagnostic algorithm performed at the TIFHC laboratory (RDT plus ELISA-rec and ELISA-lys for discordant results) consisted in determining its performance in comparison to that of the gold-standard performed at the CPHL (HAI plus ELISA-rec and IIF for discordant results). The following parameters were considered: (a) sensitivity (Se), correctly classified positive subjects; (b) specificity (Sp), correctly classified negative subjects; (c) positive predictive value (PPV), the proportion of positive patients that were really positive; (d) negative predictive value (NPV), the proportion of negative patients that were really negative; (e) diagnostic efficiency (DE), the proportion of individuals correctly classified. The test agreement, i.e., the degree of concordance or consistency between the results of the diagnostic tools, was estimated using the kappa statistic (κ) [31]. The Kappa’s statistics value has been interpreted following Cohen’s interpretation corrected by McHugh ML: values between 0–0.20 indicate no agreement, 0.21–0.39 as minimal, 0.40–0.59 as weak, 0.60–0.79 as moderate, 0.80–0.90 as strong, and above 0.90 almost perfect agreement [32].

Prevalence was calculated as the proportion of individuals with positive serological tests, using a weighted approach to adjust for the overrepresentation of seropositive individuals in the confirmatory subsample. We applied weights based on the proportions of positive and negative cases in the total screened population and the subsample of 350 confirmed individuals, ensuring the final estimate accurately reflected the total population distribution. To explore associations between individual risk factors and *T. cruzi* infection prevalence, we described and summarized participant’s baseline characteristics, including age, sex, village of origin, ethnic group, place of work, clinical variables, CD-related risk factors.

Participants’ sociodemographic characteristics were described using summary statistics. Continuous variables were summarized using mean or median (depending on the distribution of the variable) and SD or IQR. Categorical variables were described using frequencies and percentages and confidence interval at 95% (95% CI). Prevalence was described by percentage and 95% CI. Proportions were compared between groups using χ2 or Fisher’s exact tests, depending on data patterns and characteristics. Continuous variables were compared between groups using independent t test or Wilcoxon rank values according to the variables’ characteristics (whether they followed a normal distribution or not).

All variables that exhibited an association with a *p*-*value* of ≤ 0.05 from the bivariate analysis were included in the multivariate logistic regression model, with adjusted odds ratios (aORs) and 95% CI reported. In the multivariate approach, we employed a stepwise analysis method iteratively adding or removing variables based on statistical criteria, such as *p-values* of removal <0.2 and entry of <0.05, to select independent variables that significantly contribute to predicting a dependent variable. All analyses were carried out using Stata 14 (StataCorp LLC, USA) with a significance level set at *p*-value ≤ 0.05 [33].

## 3. Results

### 3.1. Participants demographics

**Fig 3:**
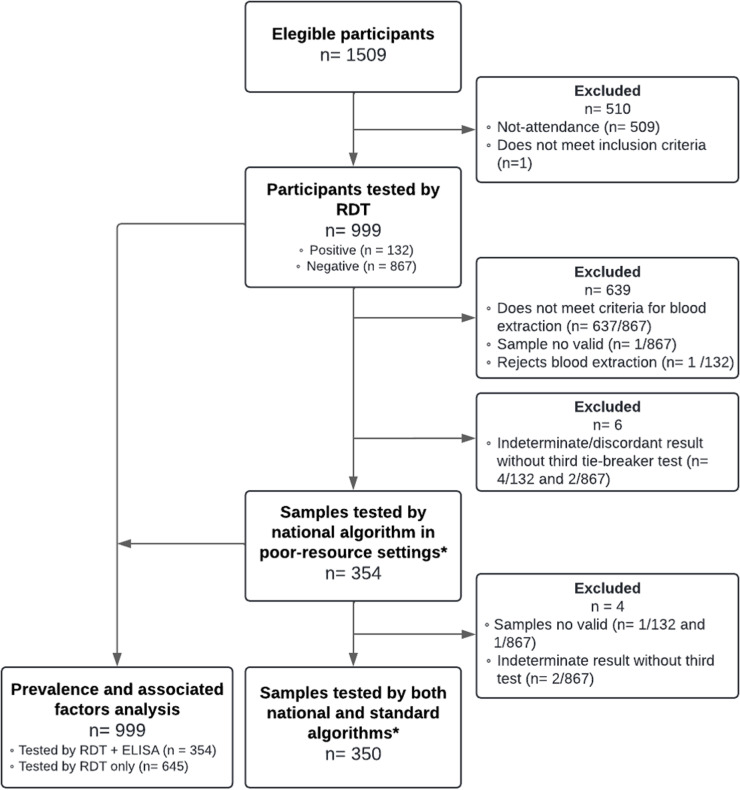
Study flowchart depicting the excluded and included participants. *National algorithm (for resource-limited settings): RDT Stat-Pak and ELISA-rec, and ELISA-lys for discordant results. Standard algorithm: HAI and ELISA-rec, and IIF for discordant results.

After applying inclusion and exclusion criteria, a total of 999 participants (66.2% of the eligible population) were recruited (Fig 3). A significant percentage of the village residents underwent screening. The distribution of participants per village, expressed as sampled population size and participation rate (in %), was: Casanillo, 517 (76.5%); San Rafael, 152 (54.3%); Campo Aroma, 151 (60.9%); Tres Palmas, 117 (79.1%); Capiatá, 55 (51.9%); Linda Vista, 4 (100%); Campo Rayo, 3 (6.4%). The median age of participants was 26 years old (IQR 12–45). The youngest participant was 1 year old and the oldest 93 years old. In total, 51.1% of the study participants were female. Regarding ethnicity, the primary ethnic groups reported were Toba-Maskoy (77.8%), Sanapaná (14.2%) and Guaná (4.9%). The remaining groups constituted 3.1% of the participants, including Angaité, Guaraní, Enxet Sur, and others (Table 1).

**Table 1:** Demographic data of study participants.

Participants demographics (N = 999)	
**Characteristics**	**Frequency, n (%)**
**Sex**	
Female	510 (51.1)
Male	489 (48.9)
**Age, median (IQR)** ^1^	
	26 (12 - 45)
**Age group** ^1^	
1–5 yrs	56 (5.6)
6–14 yrs	253 (25.4)
15–29 yrs	239 (24.0)
30–64 yrs	376 (37.8)
65 yrs or more	72 (7.2)
**Village**	
Casanillo center	517 (51.8)
San Rafael	152 (15.2)
Campo Aroma	151 (15.1)
Tres Palmas	117 (11.7)
Capiatá	55 (5.5)
Linda Vista	4 (0.4)
Campo Rayo	3 (0.3)
**Ethnicity**	
Toba-Maskoy	777 (77.8)
Sanapaná	142 (14.2)
Guaná	49 (4.9)
Other	31 (3.1)

^1^N = 996, missing data n = 3.

### 3.2. Diagnostic test performance

#### 3.2.1. Results of the national algorithm for resource-constrained settings: RDT and ELISA-rec.

The RDT result was positive for 13.2% (n = 132/999) and negative for 86.8% (n = 867/999) of tested participants. A total of 360 samples were obtained for diagnostic confirmation (Fig 3), consisting of 99.2% (n = 131/132) of those screened as positive with the RDT, and 26.4% (n = 229/867) of the RDT negative results. The ELISA-rec rendered 39.7% (n = 143/360) positive, and 58.6% (n = 211/360) negative results. Since the ELISA-rec read-out conceives a gray zone of OD values, three samples (0.83%, n = 3/360) were reported as indeterminate. Another three samples (0.83%, n = 3/360) showed discordant results in the ELISA-rec compared to the RDT. These could not be included in the subsequent analysis, as insufficient sample volume prevented the completion of the tie-breaker ELISA tests, as required for formal inclusion (Fig 3).

The RDT demonstrated a high level of concordance with the ELISA-rec test at the TIFHC laboratory, with an overall agreement of 92.6% (κ = 0.84, CI95% 0.79–0.90). In total, 26 samples (7.3%) showed discordant results between the RDT and the ELISA-rec, which were subsequently evaluated using the ELISA-lys as a tiebreaker. The ELISA-lys categorized three of these discordant samples as positive and 23 as negative. Of the 26 discordant samples, 20 (76.9%) matched the initial RDT result, reinforcing its accuracy in most cases of discordance. Ultimately, across all samples tested, 36% (n = 127/354) were classified as positive and 64% (n = 227/354) as negative.

#### 3.2.2. Results of the standard algorithm: HAI and ELISA-rec.

Of the 354 samples evaluated at the laboratory in the TIFHC, 350 were transported and processed in the CPHL (Fig 3). The agreement between HAI and ELISA-rec in the latter was almost perfect with 95.6% of agreement (κ = 0.90, 0.86–0.95). A total of 16 samples (4.6%, n = 16/350) yielded discordant results. Of these, the IIF third test confirmed seven as positive and nine as negative, to yield a final 37.1% of positive samples (n = 130/350) and 62.9% (n = 220/350) of negativity. The IIF results were 50% in agreement with the results yielded by the HAI at CPHL.

#### 3.2.3. Comparative performance of the national (RDT-based) versus PAHO/WHO standard algorithms.

We individually assessed the performance of the RDT and ELISA-rec alone and their combined performance at TIFHC laboratory compared to the standard algorithm results (HAI and ELISA-rec) obtained at the CPHL.

The analysis of performance between both diagnostic algorithms revealed an agreement of the national algorithm of 97.1% with an almost perfect kappa value (κ = 0.94, 95% CI: 0.90–0.98) (Table 2). When compared alone, the RDT agreement against the standard algorithm was 96.0% with an almost perfect kappa value (κ = 0.91, 95% CI: 0.87–0.96) and that obtained by ELISA-rec was 94.3% with a strong kappa value (κ = 0.88, 95% CI: 0.83–0.93) (Table 2).

**Table 2:** Comparison of the results obtained at TIFHC with the CPHL.

	Standard algorithm (HAI+ELISA)^*^	National algorithm (RDT+ELISA)^#^	RDT Stat-Pak	ELISA-rec
**True positive**	130	123	123	126
**False negative**	0	7	7	4
**True negative**	220	217	213	204
**False positive**	0	3	7	16
**Total**	350	350	350	350
**Se**^1^ **(95% CI)**	–	94.6% (89.2–97.8)	94.6% (89.2–97.8)	96.9% (92.3–99.2)
**Sp**^1^ **(95% CI)**	–	98.6% (96.1–99.7)	96.8% (93.6–98.7)	92.7% (88.5–95.8)
**% Agreement**	–	97.1%	96.0%	94.3%
**Kappa (95% CI)**	–	0.94 (0.90–0.98)	0.91 (0.87–0.96)	0.88 (0.83–0.93)
**PPV**^1^ **(95% CI)**	–	97.6% (93.2–99.5)	94.6% (89.2–97.8)	88.7% (82.3–93.4)
**NPV**^1^ **(95% CI)**	–	96.9% (93.7–98.7)	96.8% (93.6–98.7)	98.1% (95.1–99.5)

^1^Se, Sensitivity, Sp, Specificity, PPV, Positive Predictive Value; NPV, Negative Predictive Value.

* Standard algorithm results were obtained at CPHL upon the agreement of two conventional serological tests (HAI and ELISA-rec, both by Wiener Lab) and the use of IIF as third test if discordance between the former.

# National algorithm in resource-limited settings for serological detection of chronic *T. cruzi* infection performed at TIFHC laboratory involved Stat-Pak RDT and Wiener ELISA-rec, and the use of IICS ELISA-lys to untie if discordance between the former.

In terms of Se and Sp, the national algorithm for resource-constrained settings demonstrated a Se of 94.6% (95% CI: 89.2–97.8) and a Sp of 98.6% (95% CI: 96.1–99.7), when compared to the standard algorithm recommended by PAHO/WHO. In turn, when assessed on its own, the RDT exhibited the same Se that was retrieved when applying the RDT-based national algorithm, but slight lower Sp (96.8%, 95% CI: 93.6–98.7) than the former (Table 2). In contrast, the ELISA-rec alone showed a higher Se (96.9%, 95% CI: 92.3–99.2), yet its Sp was 92.7% (95% CI: 88.5–95.8) (Table 2).

The PPV and NPV values for the national algorithm rendered values of 97.6% (95% CI: 93.2–99.5) and 96.9% (95% CI: 93.7–98.7), respectively (Table 2). When considered individually, the RDT demonstrated a PPV of 94.6% (95% CI: 89.2–97.8), while its NPV (96.8%, 95% CI: 93.6–98.7) was almost identical to that of the complete national algorithm (Table 2). In comparison, the ELISA-rec had a lower PPV (88.7%, 95% CI: 82.3–93.4) than the complete national algorithm, and a NPV of 98.1% (95% CI: 95.1–99.5), the highest of all calculated (Table 2). Overall, the diagnostic efficacy of the national algorithm combining RDT and ELISA-rec was superior for the complete national algorithm (97.1% agreement), followed by the RDT alone (96%), and the ELISA-rec (94.3%) (Table 2)

#### 3.2.4. Assessment of the TIFHC laboratory biochemist performance with CD diagnostics.

To evaluate the TIFHC laboratory biochemist proficiency in conducting ELISAs (ELISA-rec and ELISA-lys), an external assessment focusing on the accuracy of the test’s execution was conducted by the CPHL. Out of the 354 samples run by ELISA-rec at TIFHC by the health center biochemist, 350 were re-tested at the CPHL by a different blinded operator. Four samples could not be included in this step of the analysis due to insufficient or invalid material (Fig 3). The agreement between both was 96.3% (κ = 0.92, 95% CI: 0.88–0.96). Regarding the assessment of the ELISA-lys, all 26 discordant samples were processed by ELISA IICS in both laboratories, resulting in an agreement with a kappa value of 0.78 (95% CI: 0.37–1) that indicates a poor agreement (Table 3).

**Table 3:** Assessment of the performance of the two ELISAs used at TIFHC laboratory by sample analysis with the same tests at CPHL.

ELISA Test	n	Agreement (%)	Kappa (95% CI)	Samples positive TIFHC/CPHL	Samples negative TIFHC/CPHL
**Wiener - recombinant antigen**	350	96.3%	0.92 (0.88–0.96)	134/139	203/211
**IICS – lysate antigen**	26	96.2%	0.78 (0.37–1)	2/2	23/24

### 3.3. Prevalence and associated factors of *T. cruzi* infection in the Casanillo population

**Fig 4:**
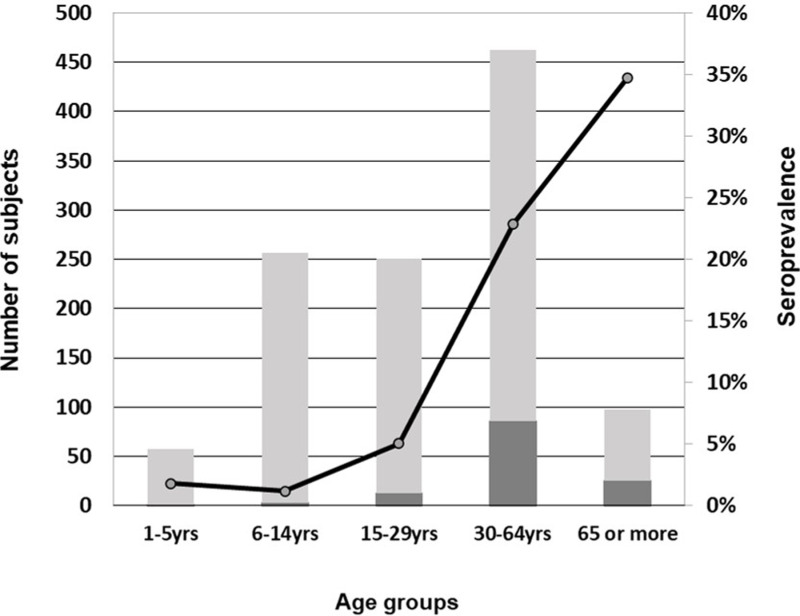
Seroprevalence distribution per age groups. Dark bars represent the count of positive samples out of the total subjects per age group (complete bars), plotted on the left Y-axis scale. The line graph illustrates the percentage of positive patients per age group, shown on the right Y-axis scale.

According to the results obtained at TIFHC, the weighted seroprevalence of *T. cruzi* infection for the total population of Casanillo was determined to be 12.6% (95% CI: 9.56–16.52). The distribution of the seroprevalence varied according to age groups, being lower in the groups aged 1–5 (1.8%, n = 1/56), 6–14 (1.2%, n = 3/253) and 15–29 years (5.0%, n = 12/239), compared to those in the 30–64 (22.9%, n = 86/376) and 65 years or older (34.7%, n = 25/72) (Fig 4). Furthermore, 5.9% (n = 59/999) of females and 8.8% (n = 68/999) of males tested seropositive, without any statistically significant difference noted in infection rates between sexes (OR 0.73, 95% CI: 0.41–1.31, *p* = 0.526). Of the 510 women who participated in this study, 269 were at childbearing age. The seroprevalence in this group was 10.8% (n = 29/269).

Among the total number of seropositive patients in our study, 80 individuals recalled having previously undergone diagnostic testing for *T. cruzi* infection. Of these 80, seven reported having tested positive for *T. cruzi* at the time of their previous examination, and three of these seven also recalled having received treatment. Notably, two of these treated individuals tested positive again in our current study.

After controlling for confounding variables, the fully adjusted model for *T. cruzi* infection suggests that age is associated with a slight increase in the likelihood (OR 1.04, 95% CI: 1.03–1.06) of *T. cruzi* infection (Tables 4 and 5). Being able to identify the CD vector showed increased odds (OR 2.43, 95% CI: 1.27–4.65) compared to those who were unaware of it. Additionally, a lower odd of infection was observed among individuals belonging to the Sanapaná (OR 0.40, 95% CI: 0.17–0.91) compared to the Toba-Maskoy ethnic group. No significant associations were detected between *T. cruzi* infection and variables such as sex, place of work/main activity, or village. Furthermore, there were no significant correlations between *T. cruzi* seropositivity and factors such as the current presence of vectors at home, a prior history of blood transfusion or donation, consumption of raw food, presence of sleeping animals in the household, or family history of CD. Additionally, none of the clinical history or habits variables studied exhibited a significant association, including diabetes mellitus, digestive disease, cardiac disease, smoking, and alcohol consumption habits (Table 4).

**Table 4:** Results for bivariable analysis of participants’ demographic, *T. cruzi* risk factors, clinical history and habits characteristics according to *T. cruzi* infection status.

Variables	*T. cruzi* infection				
	Positive, n (%)	Negative, n (%)	Total, N	n/N (%)	*p-value*
	127 (12.7)	872 (87.3)	999	100	
**Demographic characteristics**					
**Sex**					0.268
Female	59 (11.6)	451 (88.8)	508	50.9	
Male	68 (14.1)	421 (87.2)	483	48.4	
**Age in years, median (IQR)** ^1^					**<0.000**
	51 (40-62)	23 (12-40)			
**Village**					**0.048**
Casanillo center	75 (14.5)	442 (85.5)	517	51.8	
San Rafael	17 (11.2)	135 (88.8)	152	15.2	
Campo Aroma	18 (11.9)	133 (88.1)	151	15.1	
Tres Palmas	6 (5.1)	111 (94.9)	117	11.7	
Capiatá	10 (18.2)	45 (81.8)	55	5.5	
Linda Vista	0 (0.00)	4 (100)	4	0.4	
Campo Rayo	1 (33.3)	2 (66. 7)	3	0.3	
**Ethnicity**					**0.006**
Toba-Maskoy	111 (14.3)	666 (85.7)	777	77.8	
Sanapaná	7 (4.9)	135 (95.1)	142	14.2	
Guaná	4 (8.2)	45 (91.8)	49	4.9	
Other	5 (16.1)	26 (83.9)	31	3.1	
**Place of work/main activity**					**<0.000**
Agricultural	29 (18.5)	128 (81.5)	157	15.7	
House-based	51 (17.3)	244 (82.7)	295	29.5	
School	5 (2.1)	238 (97.9)	243	24.3	
Other	42 (13.8)	262 (86.2)	304	30.4	
***T. cruzi*** **infection risk factors**					
**Presence of vector at home**					0.053
Intradomicile	24 (15.8)	128 (84.2)	152	15.2	
Perdomicile	42 (15. 7)	226 (84.3)	268	26.8	
None	61 (10.5)	518 (89.5)	579	58.0	
**Previous blood transfusion**	10 (29.4)	24 (70.6)	34	3.4	**0.007**
**Blood donor**	8 (36.4)	14 (63.6)	22	2.2	**0.004**
**Consumption of raw food**	2 (12.5)	14 (87.5)	16	1.6	**1**
**Presence of animals sleeping at home**	26 (14.2)	157 (85.8)	183	18.3	0.502
**Ability to recognize the vector**	115 (16.8)	569 (83.2)	684	68.5	**<0.000**
**Family history of** ***T. cruzi*** **infection**	3 (17.6)	14 (82.4)	17	1.7	0.470
**Clinical history and habits**					
**Smoking**	29 (18.5)	128 (81.5)	157	15.7	**0.018**
**Alcohol**	19 (17.6)	89 (82.4)	108	10.8	0.107
**Diabetes mellitus**	2 (28.6)	5 (71.4)	7	0.7	0.220
**Cardiac disease**	41 (25.8)	118 (74.2)	159	15.9	**<0.000**
**Digestive disease**	18 (20.9)	68 (79.1)	86	8.6	**0.017**
**Other**	1 (5.9)	16 (94.1)	17	1.7	0.712

^1^N = 996, missing data n = 3.

**Table 5:** Results of the multivariable logistic regression model with stepwise analysis to assess the independent effects of sub-group characteristics on the risk of experiencing *T. cruzi* infection.

Variables included in the multivariate model	*T. cruzi* infection	
	(n = 127)	
	OR (95% CI)	*P-value*
**Age**	1.04 (1.03–1.06)	**<0.001**
**Awareness of the vector**	2.43 (1.27–4.65)	**0.007**
**Ethnicity**		
Toba-Maskoy	1.00 (Ref)	
Sanapaná	0.40 (0.17–0.91)	**0.030**
Other	2.41 (0.93–6.25)	0.070
**Place of work/main activity**		
Other	1.00 (Ref)	
School	0.40 (0.15–1.08)	0.071

## 4. Discussion

This study provides insight into the reliability of using an RDT (in this study Stat-Pak, Chembio, USA) for CD screening campaigns in communities with limited access to healthcare and its integration within diagnostic algorithms for rural endemic settings. Comprehensive data on the current burden of CD and its associated factors emphasize the need for continued control and prevention efforts in socioeconomically disadvantaged communities in the Paraguayan Chaco.

In Paraguay, data on the validation of serological methods are scarce [34], and as far as we know, there are no previous in-depth studies on the accuracy of RDTs in the field. However, RDTs are used routinely in screening campaigns [35] and have recently been included in the national CD Clinical Guidelines as the primary diagnostic test in resource-limited areas [10]. Among the RDTs included in the NCCP portfolio at the time of conducting this study were the Chagas Stat-Pak (Chembio Inc., USA), Chagas Antibody Test Cassette (Artron Laboratories, Canada) and the SD Bioline Chagas AB rapid test (Abbott, USA) [35]. Of these, only Stat-Pak allow direct use of capillary blood without the need for laboratory processing. Moreover, Stat-Pak demonstrated the highest performance (sensitivities and specificities > 98%) in controlled clinical studies and a meta-analysis [22,23,28]. Plus, it is the only commercialised RDT in Paraguay that was previously validated in community-based screening campaigns in the Bolivian Chaco and in rural areas of Colombia [25,36].

Our results on the use of Stat-Pak as a single method showed a good sensitivity (Se, 94.6% [95% CI: 89.2–97.8]) and specificity (Sp, 96.8% [95% CI: 96.1–99.7]) values. In a similar study in Bolivian Chaco, Lozano and co-workers reported a punctual Se and Sp of 97.7% and 97.4%, respectively (25), both of which fall within our 95% CI. Variations in the RDT accuracy between both studies may be influenced by genetic, ecological and operational characteristics associated with non-controlled environments encountered in screening campaigns in the field.

A recent systematic review on the diagnostic accuracy of ELISAs and RDTs observed that the overall sensitivity of the RDTs is lower than that of the ELISAs [22]. According to Porras and co-workers [37], the lower Se of the RDTs compared to the ELISAs might render the RDTs unsuitable as a stand-alone test for diagnosing CD. However, in this study, the Se of the RDT (94.6% [95% CI: 89.2–97.8]) compared to that of the ELISA-rec (96.9% [95% CI: 92.3–99.2]) suggests that the RDT may not be less sensitive as stand-alone test since their confidence intervals overlap. Taking this into consideration, with the prevalence of 12.6% (95% CI: 9.56–16.52) of the 999 participants observed in this study, relying on the RDT outcome alone would have result in the description of 7 false negative and 26 false positive detections, thus leading to a 0.7% misdiagnosis of true positive subjects, and unnecessarily treating 2.6%. Although the slightly higher accuracy achieved with the current integrated national diagnosis approach (κ = 0.94 [95% CI: 0.90–0.98] and κ = 0.91 [95%CI: 0.87–0.96]), the false negative detection rate of using a single RDT as a diagnostic tool would remain below 1%. Moreover, the higher agreement of the RDT in this study (96% versus 94.3% of ELISA-rec), along with the comparable Se and Sp to the national algorithm, further demonstrate the suitability of Stat-Pak for inclusion in diagnostic protocols in the Paraguayan Chaco, though its high cost could limit its accessibility and scalability for widespread in routine use. To address this limitation, it would be beneficial to explore other options, such as conducting validation studies for locally produced test that might offer more affordable alternatives [34].

The diagnostic algorithm for resource-limited settings that integrates RDTs and ELISAs, as contemplated in Paraguay national CD Clinical Guidelines, showed a notable diagnostic concordance (97% agreement) with the standard algorithm used at a reference laboratory fully relying on conventional serological tools, suggesting that only three out of 100 individuals may be misdiagnosed. Its high Se and Sp (94.6% [95% CI: 89.2–97.8] and 98.6% [95% CI: 96.1–99.7], respectively), along with its almost perfect reliability values (k = 0.94 [95% CI: 0.90–0.98]), indicate its validity for inclusion in the diagnostic algorithm for CD in areas with limited laboratory resources and personnel, such as the Paraguayan Chaco.

When assessing the performance of the ELISAs in the two laboratories, very good agreement rates were retrieved. The chance to count in-place with a third test, if needed, has major advantages for laboratories located in distant rural areas like the Chaco region. Bearing in mind the distance to the reference laboratory at Asuncion, the costs and risks of shipping samples, and the delay in the turnaround of results to the community, diagnostic independence should be encouraged as much as possible in such peripheral facilities.

Our results suggest that the potential use of RDTs, as stand-alone tests or in combination with an ELISA test, as a routine diagnostic strategy should be further explored. Given the antigenic diversity of *T. cruzi* and the genetic variability within human populations, geographical validation of serological tools (be it conventional or RDTs) is highly advisable before their mass deployment [34,38]. Additionally, crucial environmental, operational, and cultural factors need also to be considered. Logistical challenges, such as long distances, poor road conditions, and limited accessibility exacerbated by adverse weather, where rain can render roads and paths almost impassable, underscore the complexity of deploying diagnostic tools in such remote settings. Moreover, cultural concerns and the ethnographic context of the community play a significant role in shaping perceptions of healthcare access, further influencing the acceptance and effectiveness of diagnostic strategies. Considering that RDTs are particularly well-suited for resource-constrained settings, and their performance can be equivalent to conventional tools or even show improved diagnostic efficacy, a more ambitious implementation by national health systems should be pursued in those contexts at least. Adopting regionally tailored algorithms based on RDTs with proven high performance could improve the access to diagnosis for thousands of people in isolated regions. This is especially meaningful in the current context where the diagnostic recommendations for CD are being revised by the international scientific community [7,23–25,39].

This study validated the use of RDTs in a highly endemic, remote rural area with limited diagnostic capacity, demonstrating their suitability for field screening in resource-constrained settings. In Paraguay, the national algorithm incorporating RDTs is specifically designed for such isolated regions and is not intended for universal applicability [10]. However, the use of RDTs is also being evaluated in other regions of Paraguay with lower endemicity and greater diagnostic capacity as part of the Unitaid-funded “Cuida Chagas” project [35]. This initiative, focused on improving access to diagnosis and treatment for women of reproductive age and their newborns, uses RDTs for systematic screening across diverse municipalities and provides additional data on Stat-Pak RDT performance under varying conditions. By integrating the findings from our study with the results of this initiative, a more comprehensive understanding of Stat-Pak’s performance and its feasibility for broader use in Paraguay will emerge. Nevertheless, the debate over using RDTs as an initial diagnostic test is likely to persist in contexts where laboratory access is readily available, underscoring the importance of tailoring diagnostic strategies to local needs and resources.

Furthermore, we would like to emphasize that for the sustainable integration of the use of RDTs, or any other innovative diagnostic test, into the healthcare system, once the test accuracy has been reassured, would entail strengthening to make sure it will adopt and embrace the innovation (be it in the shape of a tool or a process, or both). In this regard, several challenges were already encountered in this study, which reflect the daily realities faced by the healthcare system in Paraguay. These included: maintaining a consistent supply of RDTs, managing their costs, ensuring the availability of trained healthcare staff and providing CD patients with access to adequate care and follow-up. All those limitations are commonly found, and particularly pronounced, within regions affected by NTDs, undermining the effectiveness of conventional diagnostic tests, regardless of their type [4,7,17,26].

Our study found a seroprevalence of 12.6% (95% CI: 9.56–16.52) in Casanillo, significantly higher than the national estimate of 2.1% [9]. Nevertheless, that prevalence is lower than that found in surveys from the 1970s to 1990s in other indigenous populations of the same region, which showed *T. cruzi* seropositivity peaks up to 73% and domestic infestation ranging 20%–60% [13,16]. Subsequent vector control campaigns led to a considerable reduction in the proportion of infested houses nationally [16]. The lower infection rates observed in current studies within the Gran Chaco populations align with socioeconomic and cultural differences among communities [40,41] contributing to disparities in *T. cruzi* seroprevalence despite shared ecological environment.

Aligned with the vector control efforts, the observed seroprevalence significantly increased in age groups >15 years old. The higher rate among older individuals is the consequence of cumulative exposure to vector-mediated transmission at a time when vector control had not been established. Conversely, infection among individuals under 15 years old indicates either vertically acquired events or recent vector-borne transmission [42,43]. Paraguay was certified free of *T. cruzi* vector transmission by the domiciled *T. infestans* triatomine species in 2018 [8]. Therefore, younger study participants might have been marginally exposed to triatomine vectors. In fact, the last capture of triatomines and subsequent vector control measures in the Casanillo community took place in June 2022, when peridomestic vectors were detected, including four that were infected with *T. cruzi*. Moreover, of the four *T. cruzi* infection cases detected within the youngest age groups (0–14 years old), two siblings had both their parents who tested positive, and in three of the four registered cases (including those two siblings) it was reported that animals were sleeping indoors. In the fourth case, a 5-year-old girl and the youngest positive detection among all study participants, no risk factors were reported by the family, and her mother was confirmed negative through conventional serology. Altogether, this might indicate that, even if community housings were apparently not vector-infested, transmission mediated by other peridomestic or even sylvatic vector species cannot be disregarded [16]. Furthermore, the presence of seropositivity in the mother of some infant CD cases raises the possibility of vertical transmission as a contributing factor to *T. cruzi* infection acquisition in the area. When focusing on the latter, we found that about 10.8% of women at childbearing age tested positive for CD, which underscores the need to strengthen screening and prevention efforts, prioritizing maternal and infant health within CD control policies in the Chaco region [43–46].

Indigenous communities are particularly vulnerable due to cultural and linguistic uniqueness, social marginalization, housing conditions, community distances, limited access to healthcare services and varied socio-cultural knowledge about the disease [13,17,47]. Studies suggest that few individuals receive information about CD, and many are unaware of the relationship between *T. infestans* and CD [40]. Being able to recognize the vector was a statistically significant risk for CD in our study. The high exposure to CD information due to prior IEC activities focused on disease knowledge, prevention, and treatment in Casanillo led to a 60% knowledge rate about it. At the same time, no association was found between *T. cruzi* infection and vector presence at home. This suggests that knowledge about the vector might be more related to continuous experience and familiarity with the insect in their daily environment, which could have a greater influence than the mere presence of the vector in their dwellings at a given moment. However, further research is needed to evaluate how this knowledge translates into prevention measures and its long-term impact on disease incidence.

Furthermore, belonging to the Sanapaná ethnic group appears to be a protective factor against *T. cruzi* infection compared to Toba-Maskoy. Further investigation into cultural practices, social dynamics, and environmental conditions unique to each group will be needed to understand this finding. The lack of statistical significance for further factors commonly associated with CD in our study, such as the presence of animals in dwellings or a history of blood transfusion or organ transplant, suggest other variables may be more influential in determining the disease prevalence. While effective vector control measures were once a key factor in reducing disease transmission, their impact may now be less pronounced. The historically limited access to healthcare in the Chaco region, specially within indigenous populations, may have led to past vector control and diagnostics campaigns without accompanying treatment strategies, or cases where patients did not meet treatment criteria [8,47]. Moreover, factors like delayed diagnosis, inadequate treatment coverage, and the slow process of seroconversion may also contribute to persistent seropositivity in the community [16–18,38]. To better understand the unique transmission dynamics of CD within communities like Casanillo, further research is needed to explore additional risk factors, including socioeconomic conditions and healthcare accessibility, and to develop more targeted interventions.

Our assessment of the diagnostic algorithm’s field viability faces several limitations. Firstly, our study implicitly relies on the assumption of a theoretical perfect diagnostic accuracy for the reference standard algorithm. Due to resource constraints, we were unable to independently validate its accuracy. Reliance on the acknowledged gold-standard may limit the precision of our findings, as any undetected deviation of its performance would affect the diagnostic accuracy estimates. Geographic variations in assay performance present challenges to the generalizability of results. In addition, the sample includes all positive cases but only one-third of the negative cases, thus possibly underestimating sensitivity and overestimating specificity of the test within the population. Besides, despite experienced field personnel reading RDTs, inconsistencies may have arisen due to operator variability. Moreover, despite efforts in sample storage and transport, the geographical remoteness of the study site and the need for periodic batch deliveries to the reference laboratory introduced logistical challenges, such as extended transportation times and environmental factors, that might have compromised sample integrity in some cases. Lastly, regarding the prevalence and associated factors assessment, the “call effect” of our screening campaign may have introduced variability affecting the study population, challenging the precision of disease prevalence estimates.

## 5. Conclusion

Our study results provide a valuable insight into the utility and reliability of the diagnostic algorithm proposed by the Paraguayan authorities, i.e., combining RDT with classic serological methods, for diagnosing CD in resource-limited settings. The comprehensive evaluation of this algorithm against the currently recommended gold standard highlights its potential as a valuable tool for screening and clinical routine. However, our findings also underscore the need for continued validation and refinement to enhance its accuracy and reliability. Additionally, our findings on the seroprevalence of *T. cruzi* highlight that CD is a major public health problem in the Paraguayan Chaco. Considering the historically high prevalence of household infestation by vector insects and the epidemiological trend skewed toward older populations, it becomes clear that, after achieving effective vector control, the next critical step in epidemiological management is to ensure appropriate access to diagnostic and treatment, prioritizing all women of childbearing age to interrupt vertical transmission and infected infants.

## Supporting information

S1 Checklist STARD 2015 Checklist: Items based on the STARD 2015 guidelines for reporting diagnostic accuracy studies considered in this study. (DOCX)
